# Progress and Challenges in Using Oral Cholera Vaccines to Control Outbreaks: The Médecins Sans Frontières Experience

**DOI:** 10.1093/infdis/jiy487

**Published:** 2018-09-14

**Authors:** Iza Ciglenecki, Andrew S Azman, Christine Jamet, Micaela Serafini, Francisco J Luquero, Jean-Clement Cabrol

**Affiliations:** 1 Médecins Sans Frontières, Geneva, Switzerland; 2 Department of Epidemiology, Johns Hopkins University, Baltimore, Maryland; 3 Epicentre, Paris, France

**Keywords:** oral cholera vaccines, cholera, epidemics

## Abstract

The use of oral cholera vaccine (OCV) has increased since 2011, when Shanchol, the first OCV suitable for large-scale use, became available. Médecins Sans Frontières considers OCVs an essential cholera outbreak control tool and has contributed to generating new evidence on OCV use in outbreaks. We showed that large-scale mass campaigns are feasible during outbreaks, documented high short-term effectiveness and showed that vaccines are likely safe in pregnancy. We found that a single-dose regimen has high short-term effectiveness, making rapid delivery of vaccine during outbreaks easier, especially given the on-going global vaccine shortage. Despite progress, OCV has still not been used widely in some of the largest recent outbreaks and thousands of cholera deaths are reported every year. While working towards improving our tools to protect those most at-risk of cholera, we must strive to use all available effective interventions in efficient ways, including OCV, to prevent avoidable deaths today.

The use of killed oral cholera vaccine (OCV) has increased since 2011, when Shanchol, the first OCV suitable for large-scale use, became available and was prequalified by the World Health Organization (WHO) [1]. The creation of an OCV stockpile in 2013, together with funding for the vaccines and implementation costs, further facilitated vaccine use. Doubts about feasibility, timeliness, and acceptability of OCV, and the fear of diverting resources from other preventive interventions, which predominated discussions and discouraged reactive OCV use in the past [2], have been mostly put to rest by successful OCV field implementations. However, timeliness of reactive OCV campaigns, the logistic complexities of delivering the vaccine, and the persistent global shortage continue to hinder widespread OCV use.

Médecins Sans Frontières considers OCV to be an essential outbreak control tool, complimenting case management and water/sanitation/hygiene interventions. In 2012, we assisted the Guinea Ministry of Health in organizing a reactive vaccination campaign, targeting over 200 000 people. We demonstrated that reactive mass campaigns are feasible, and that even in remote rural villages high coverage can be reached (eg, >90% having received at least 1 dose and 76% 2 doses) [3, 4]. We documented high short-term vaccine effectiveness (86.6%; 95% confidence interval [CI], 56.7%–95.8%) and showed that the vaccine is likely safe in pregnancy [5, 6].

One important challenge for reactive use of current OCVs is the recommended 2-dose schedule. With evidence that a single OCV dose provides some short-term protection at the individual level [7] and may prevent more cases in an outbreak than providing 2 doses to half of the people [8], we assisted the Ministry of Health in Juba, South Sudan with adopting this single-dose strategy during a 2015 epidemic when only limited OCV was available. We demonstrated high short-term single-dose vaccine effectiveness of 87.3% (95% CI, 70.2%–100%) [9]. Less than a year later, when cholera broke in Zambia’s capital Lusaka, after several years of absence, a similar situation involving a large urban population at risk and limited amount of vaccines appeared. With the same 1-dose strategy we reached almost half a million people, and were able to confirm the short-term effectiveness of this regimen (88.9%; 95% CI, 42.7%–97.8%) in a population where cholera had not been reported for more than 4 years [10]. As it is unclear how long protection from a single dose may last, the campaign was followed with a second dose 8 months later, when more vaccine was available. Current immunologic evidence suggests that the recommended 14-day interval is suboptimal, though more work is needed to define when booster doses should be provided [11].

Although OCVs are known to be thermostable, the vaccine package inserts require storage in the cold chain. This, coupled with the bulkiness of the vaccines, presents a major logistic challenge in organizing large-scale campaigns. In agreement with respective ministries of health, we opted for off-label vaccine use in most of our campaigns. Taking advantage of demonstrated OCV heat stability [12], we kept vaccines stored in the central cold chain, but did not use passive cold chain (ie, cold boxes and vaccine carriers) on the day of vaccination, greatly reducing cold chain needs and related resources and costs. The above described vaccine effectiveness estimates were obtained from campaigns using this out-of-cold-chain strategy. Our example has so far not been widely followed, and most of the other OCV campaigns, even in the countries with previous experience of using the vaccines out-of-cold-chain, have been conducted with strict cold chain use [5, 9, 10]. Fortunately, Shanchol recently received WHO prequalification for controlled cold chain use, allowing the vaccine to be used with up to 14 days at ambient temperature not exceeding 40°C [13], and its bioequivalent, Euvichol, expected to get approval soon.

Optimal reactive use of OCV is hindered by delays in recognizing, confirming, and declaring cholera epidemics [14]. Improving surveillance systems will be essential in fight against cholera. But many of the recent large nationwide epidemics were protracted and even delayed campaigns would save many lives. More cholera cases have been reported to the WHO in 2017 than in any other year since the start of the seventh cholera pandemic, with unprecedented epidemics in war-torn Yemen and in eastern and central Africa [15, 16]. With only a few exceptions, despite great advances, OCVs have not been used in the major epidemics of 2017. The shortage of vaccines remains the biggest obstacle, even with recent increases in production. It forces health authorities to choose which populations will benefit from the vaccine and which cannot—a particularly difficult task with large populations at risk. Even if allocation decisions are epidemiologically justifiable, they may not be politically acceptable. Alternative, more targeted strategies, such as those focused on neighbors of cases [17, 18] or mobile high-risk groups [19], are rarely explored. Not every cholera outbreak is the same: an evidence-based “menu” of standardized intervention packages based on local epidemiology could greatly facilitate decision-making processes.

While long-term cholera control or elimination will likely depend on tackling cholera in areas with persistent transmission, it is during epidemics that the cholera burden is most devastating [20]. In the 21st century, with means available to both prevent and treat cholera, we should not accept thousands of cholera deaths reported every year. OCVs might not be a perfect outbreak response tool, but we must strive to use all available effective interventions and explore innovative ways to deliver them to those most at risk to prevent avoidable deaths today.

## Notes

***Supplement sponsorship.*** This work is part of a supplement sponsored by the Bill and Melinda Gates Foundation.

***Potential conflicts of interest.*** A. S. A. reports grants from Bill and Melinda Gates Foundation, outside the submitted work; F. J. L. reports grants, personal fees, and nonfinancial support from Médecins sans Frontières, personal fees and nonfinancial support from DOVE project, and nonfinancial support from Institut Pasteur, outside the submitted work. All other authors report no potential conflict. All authors have submitted the ICMJE Form for Disclosure of Potential Conflicts of Interest. Conflicts that the editors consider relevant to the content of the manuscript have been disclosed.

## References

[1] Pezzoli L, Oral Cholera Vaccine Working Group of the Global Task Force on Cholera Control Deployments from the oral cholera vaccine stockpile, 2013–2017. Wkly Epidemiol Rec 2017; 92:437–42. 28799734

[2] Date KA, Vicari A, Hyde TB, et al Considerations for oral cholera vaccine use during outbreak after earthquake in Haiti, 2010-2011. Emerg Infect Dis 2011; 17:2105–12. 22099114 10.3201/eid1711.110822 PMC3310586

[3] Ciglenecki I, Sakoba K, Luquero FJ, et al Feasibility of mass vaccination campaign with oral cholera vaccines in response to an outbreak in Guinea. PLoS Med 2013; 10:e1001512. 24058301 10.1371/journal.pmed.1001512 PMC3769208

[4] Luquero FJ, Grout L, Ciglenecki I, et al First outbreak response using an oral cholera vaccine in Africa: vaccine coverage, acceptability and surveillance of adverse events, Guinea, 2012. PLoS Negl Trop Dis 2013; 7:e0002465. 10.1371/journal.pntd.0002465 PMC3798604 24147164

[5] Luquero FJ, Grout L, Ciglenecki I, et al Use of Vibrio cholerae vaccine in an outbreak in Guinea. N Engl J Med 2014; 370:2111–20. 24869721 10.1056/NEJMoa1312680

[6] Grout L, Martinez-Pino I, Ciglenecki I, et al Pregnancy outcomes after a mass vaccination campaign with an oral cholera vaccine in Guinea: a retrospective cohort study. PLoS Negl Trop Dis 2015; 9:e0004274. 26713614 10.1371/journal.pntd.0004274 PMC4695076

[7] Qadri F, Wierzba TF, Ali M, et al Efficacy of a single-dose, inactivated oral cholera vaccine in Bangladesh. N Engl J Med 2016; 374:1723–32. 27144848 10.1056/NEJMoa1510330

[8] Azman AS, Luquero FJ, Ciglenecki I, Grais RF, Sack DA, Lessler J The impact of a one-dose versus two-dose oral cholera vaccine regimen in outbreak settings: a modeling study. Plos Med 2015; 12:e1001867. 26305226 10.1371/journal.pmed.1001867 PMC4549326

[9] Azman AS, Parker LA, Rumunu J, et al Effectiveness of one dose of oral cholera vaccine in response to an outbreak: a case-cohort study. Lancet Glob Health 2016; 4:e856–63. 27765293 10.1016/S2214-109X(16)30211-X

[10] Ferreras E, Chizema-Kawesha E, Blake A, et al Single-dose cholera vaccine in response to an outbreak in Zambia. N Engl J Med 2018; 378:577–9. 29414267 10.1056/NEJMc1711583

[11] Matias WR, Falkard B, Charles RC, et al Antibody secreting cell responses following vaccination with bivalent oral cholera vaccine among Haitian adults. PLoS Negl Trop Dis 2016; 10:e0004753. 27308825 10.1371/journal.pntd.0004753 PMC4911095

[12] Saha A, Khan A, Salma U, et al The oral cholera vaccine Shanchol™ when stored at elevated temperatures maintains the safety and immunogenicity profile in Bangladeshi participants. Vaccine 2016; 34:1551–8. 26896684 10.1016/j.vaccine.2016.02.020

[13] World Health Organization. WHO prequalified vaccines. Cholera: inactivated oral Shanchol, 2018 https://extranet.who.int/gavi/PQ_Web/PreviewVaccine.aspx?nav=0&ID=249. Accessed 11 August 2018.

[14] Parker LA, Rumunu J, Jamet C, et al Adapting to the global shortage of cholera vaccines: targeted single dose cholera vaccine in response to an outbreak in South Sudan. Lancet Infect Dis 2017; 17:e123–7. 28109819 10.1016/S1473-3099(16)30472-8

[15] World Health Organization. Weekly bulletin on outbreaks and other emergencies: WHO Africa Regional Office, 2018 http://apps.who.int/iris/bitstream/10665/259809/1/OEW1-2018.pdf. Accessed 11 August 2018.

[16] World Health Organization. Weekly Epidemiological Monitor - Eastern Mediterranean Regional Office, February2018 http://applications.emro.who.int/docs/epi/2018/Epi_Monitor_2018_11_07.pdf?ua=1. Accessed 11 August 2018.

[17] Parker LA, Rumunu J, Jamet C, et al Neighborhood-targeted and case-triggered use of a single dose of oral cholera vaccine in an urban setting: Feasibility and vaccine coverage. PLoS Negl Trop Dis 2017; 11:e0005652. 28594891 10.1371/journal.pntd.0005652 PMC5478158

[18] Finger F, Bertuzzo E, Luquero FJ, et al The potential impact of case-area targeted interventions in response to cholera outbreaks: A modeling study. PLoS Med 2018; 15:e1002509. 29485987 10.1371/journal.pmed.1002509 PMC5828347

[19] Heyerdahl LW, Ngwira B, Demolis R, et al Innovative vaccine delivery strategies in response to a cholera outbreak in the challenging context of Lake Chilwa. A rapid qualitative assessment. Vaccine [published online ahead of print 7 November, 2017]. doi: 10.1016/j.vaccine.2017.10.108. PMC6189868 29126808

[20] Luquero FJ, Rondy M, Boncy J, et al Mortality rates during cholera epidemic, Haiti, 2010–2011. Emerg Infect Dis 2016; 22:410–6 26886511 10.3201/eid2203.141970 PMC4766911

